# Potential of Circulating Tumor DNA in Stratifying Patients with Localized pMMR Colon Cancer to Neoadjuvant Therapy

**DOI:** 10.1245/s10434-026-19539-8

**Published:** 2026-04-03

**Authors:** Henry G. Smith, Tenna V. Henriksen, Claus Lindbjerg Andersen

**Affiliations:** 1 Abdominalcenter K, Copenhagen University Hospital – Bispebjerg and Frederiksberg, Copenhagen, Denmark; 2 Department of Clinical Medicine, University of Copenhagen, Copenhagen, Denmark; 3 Department of Molecular Medicine, Aarhus University Hospital, Aarhus, Denmark; 4 Department of Clinical Medicine, Aarhus University, Aarhus, Denmark; 5 Regional Hospital Randers, Randers, Denmark; 6 Odense University Hospital, Odense, Denmark; 7 Clinical Cancer Research Center, Aalborg University, Aalborg, Denmark; 8 Regional Hospital Viborg, Viborg, Denmark; 9 Copenhagen University Hospital – Herlev, Herlev, Denmark; 10 Zealand University Hospital, Roskilde, Denmark; 11 Copenhagen University Hospital – Bispebjerg, Copenhagen, Denmark; 12 Regional Hospital Hjørring, Hjørring, Denmark; 13 Odense University Hospital, Svendborg, Denmark; 14 Aarhus University Hospital, Aarhus, Denmark; 15 Regional Hospital Horsens, Horsens, Denmark

## Abstract

**Introduction:**

Neoadjuvant chemotherapy has potential benefit in patients with localized colon cancer with proficient mismatch repair (pMMR). However, patient selection is a major challenge. We explored whether pre-operative circulating tumor DNA (pre-op ctDNA) would be helpful in selecting high-risk patients for neoadjuvant chemotherapy.

**Methods:**

Pre-op ctDNA levels were determined using blood samples from patients undergoing potentially curative surgery for localized pMMR colon cancer. Associations between pre-op ctDNA and pathological stage, positive (R1) resection margins and disease recurrence were investigated. Predictive models for these outcomes using routinely available clinical factors with and without pre-op ctDNA were developed.

**Results:**

In total, 979 patients were included. Pre-op ctDNA was significantly associated with pathological stage, although no difference between patients with stage II and III disease was noted (positive patients: stage I 28% versus stage II 67% versus stage III 75%, *p* < 0.001). Pre-op ctDNA was also associated with R1 resection (positive patients: R0 59% versus R1 82%, *p *= 0.017). Patients with positive pre-op ctDNA had increased 3-year cumulative incidence of recurrence after surgery (23% [95% CI 18–28] versus 5.3% [95% CI 2.6–9.4]; *p *< 0.001), most of which occurred within 12 months of surgery. Although the addition of ctDNA to clinical predictive models did not improve prediction of pathological stage or R1 status, it had some value in identifying early recurrence after surgery.

**Conclusion:**

Pre-op ctDNA is associated with pathological stage, R1 resection, and recurrence after surgery in patients with localized pMMR colon cancer. However, its value in improving the selection of high-risk patients for neoadjuvant chemotherapy requires further investigation.

**Supplementary Information:**

The online version contains supplementary material available at 10.1245/s10434-026-19539-8.

Most patients diagnosed with colon cancer present with localized and therefore potentially curable disease. In these patients, surgical resection remains the standard treatment, which can be supplemented with adjuvant chemotherapy in those with pathological risk factors.^1^ Despite this initial treatment, recurrent disease develops in up to 30% of patients.^2–4^ As such, an argument can be made for the need to intensify treatment in high-risk patients. Neoadjuvant chemotherapy is one potential option that has been shown to significantly reduce the risks of recurrence in other gastrointestinal cancers.^5,6^

Several recent trials have investigated the potential benefits of neoadjuvant chemotherapy in patients with locally advanced colon cancer. The PRODIGE 22 and NeoCol studies both demonstrated pathological downstaging after neoadjuvant chemotherapy but failed to show any significant differences in survival outcomes.^7,8^ A similar effect was found in the larger FOxTROT study, which also demonstrated associated reductions in the risks of incomplete resection, as well as reductions in the risks of residual or recurrent disease within 2 years of surgery.^9^ Another key finding of the FOxTROT study was the lack of benefit in patients with deficient mismatch repair (dMMR) colon cancers, who have subsequently been shown to be exquisitely sensitive to neoadjuvant immunotherapy in other studies.^10–12^

Whilst these studies suggest some benefit of neoadjuvant chemotherapy in patients with proficient MMR (pMMR) locally advanced colon cancer, they also highlighted major issues with patient selection. Inclusion to these studies was based on computed tomography (CT)-based clinical staging, which is known to have limited accuracy.^13^ This is reflected in the high rates of overstaging of up to 33% in the studies’ control arms.^7,9^ As such, the use of clinical staging to allocate patients to neoadjuvant chemotherapy would result in substantial overtreatment of low-risk patients, who would not fulfil current criteria for adjuvant therapy.

If neoadjuvant chemotherapy is to have an established role in the treatment of patients with colon cancer, more robust methods for patient stratification are needed. Circulating tumor DNA (ctDNA) has already demonstrated its potential as a versatile biomarker, facilitating a more nuanced approach to adjuvant therapy and follow-up in this patient group.^14–16^ However, its potential to identify high-risk patients before surgery is yet to be determined. We investigated the utility of pre-operative ctDNA in predicting pathological stage, resection margin status, and the risks of recurrence in patients with primary, localized colon cancer.

## Methods

### Patients

Patients with colon cancer with localized disease were recruited at 13 Danish surgical departments in the period 2016–2023. No patients received neoadjuvant chemotherapy. All patients were treated with curative intent according to national guidelines and monitored with standard-of-care CT scans at 12 and 36 months after surgery. Information on clinical risk factors was extracted from the Danish Colorectal Cancer Group quality database. Only patients with pMMR disease were included in the analysis.

### ctDNA Detection

The ctDNA detection data included in this study have been published elsewhere in their own right along with detailed methods description. Pre-operative samples, collected within a month before surgery (median 5 days, interquartile range [IQR] 3–7 days before surgery) were included. Blood samples were collected in K2-EDTA 10 mL tubes (Becton Dickinson), and plasma was isolated within 2 h by double centrifugation (10 min at 3000 g). The cell-free DNA (cfDNA) was purified from 8 mL of plasma using the QIAamp Circulating Nucleic Acids kit (Qiagen). The median cfDNA yield was 15,279 copies (IQR 10,725–22,751), all of which was used for input to ctDNA analyses. Briefly, ctDNA analysis was conducted using either tumor-informed droplet digital polymerase chain reaction (ddPCR) targeting a single, clonal variant^17^ tumor-informed panel sequencing of the 12 most commonly mutated genes in CRC,^18^ or tumor-agnostic ddPCR analysis of three CRC-specific methylation markers.^19^

### Prediction Models

The cohort was split 80:20 in a training dataset and validation dataset with the aim to balance recurrence status, performance status, clinical staging, sex, age, ctDNA detection, and tumor localization. To predict pathological staging, resection margin status, and recurrence status, respectively, logistic regression models were generated on the training data, including the following the following variables: age (>70 vs ≤70 years), sex‚ cN category, cT category (cT0–2 vs cT3 vs cT4), World Health Organization (WHO) performance (score 0–2 vs 3), screening status, and tumor localization (right vs left colon). Clinical staging (cT and cN) was performed by radiologists at the participating institutions, who were blinded to the results of ctDNA analyses. Additional models were generated with the addition of either ctDNA detection status (undetected vs detected) or ctDNA level (undetected, low level [<3 copies/mL], high level [≥3 copies/mL]). The cut-offs for high and low levels of ctDNA were determined by the median value in the positive ctDNA group, which was 2.95 copies/mL. Each model was used on the validation data and evaluated using receiver operating characteristic (ROC) curves, area under the ROC curve (AUC), and precision-recall curves. Additionally, multivariable Fine–Gray competing risk regression models were used to predict the cumulative incidence of recurrence, with death as a competing event. Patients were censored at the end of follow-up. These models were evaluated using the same input variables as the logistic regression models and on the same 80:20 split dataset for training and validation.

### Statistics

Differences in distribution of ctDNA positive patients in different groups were tested with a chi-squared test. The ctDNA level was compared between pathological Union for International Cancer Control stages, resection completion, and recurrence status using a Wilcoxon signed rank test. The cumulative incidence of recurrence was compared between ctDNA-positive and -negative patients using an Aalen–Johansen estimator, treating recurrence as an event and death as the competing event. Patients were censored at the end of follow-up. All statistical calculations were done in R version 4.2.1.

## Results

A total of 979 patients were included in the study and then divided into training (*n* = 784) and validation (*n* = 195) cohorts. The clinicopathological demographics of these patients are summarized in Table 1. The median follow-up for the entire cohort was 30 months (IQR 12–36).

**Table 1:** Clinicopathological demographics of the patient cohort

Characteristic	Overall, *N* = 979	Training, *N* = 784	Testing, *N* = 195	*p* Value
Sex				> 0.9
Female	409 (42)	328 (42)	81 (42)	
Male	570 (58)	456 (58)	114 (58)	
Age	71 (64–77)	71 (64–77)	70 (62–77)	0.6
Site				0.030
Right colon	513 (52)	397 (51)	116 (59)	
Left colon	466 (48)	387 (49)	79 (41)	
cT category				> 0.9
cT0	3 (0.3)	3 (0.4)	0 (0)	
cT1	96 (9.8)	78 (9.9)	18 (9.2)	
cT2	385 (39)	307 (39)	78 (40)	
cT3	430 (44)	342 (44)	88 (45)	
cT4	65 (6.6)	54 (6.9)	11 (5.6)	
cN category				> 0.9
cN0	595 (61)	477 (61)	118 (61)	
cN1	298 (30)	239 (30)	59 (30)	
cN2	86 (8.8)	68 (8.7)	18 (9.2)	
pUICC				> 0.9
I	227 (23)	183 (23)	44 (23)	
II	478 (49)	382 (49)	96 (49)	
III	274 (28)	219 (28)	55 (28)	
Risk				0.6
Low	835 (85)	671 (86)	164 (84)	
High	144 (15)	113 (14)	31 (16)	
Screening				0.8
Not screening-detected	674 (69)	538 (69)	136 (70)	
Screening-detected	305 (31)	246 (31)	59 (30)	
Performance status				0.6
0	736 (76)	590 (76)	146 (76)	
1	181 (19)	148 (19)	33 (17)	
2	45 (4.6)	33 (4.2)	12 (6.2)	
3	9 (0.9)	7 (0.9)	2 (1.0)	
Not available	8	6	2	
Resection completion				0.5
R0	937 (96)	753 (96)	184 (95)	
R1	37 (3.8)	28 (3.6)	9 (4.7)	
Not available	5	3	2	
Follow-up time (months)	30 (12–36)	29 (12–36)	35 (12–36)	> 0.9
Recurrence				> 0.9
No recurrence	707 (87)	566 (87)	141 (87)	
Recurrence	107 (13)	86 (13)	21 (13)	
Not available	165	132	33	
ctDNA				0.4
Negative	398 (41)	313 (40)	85 (44)	
Positive	581 (59)	471 (60)	110 (56)	

Data are presented as n (%) or median (interquartile range) unless otherwise indicated

ctDNA, circulating tumor DNA; IQR, interquartile range; UICC, Union for International Cancer Control

**Fig. 1:**
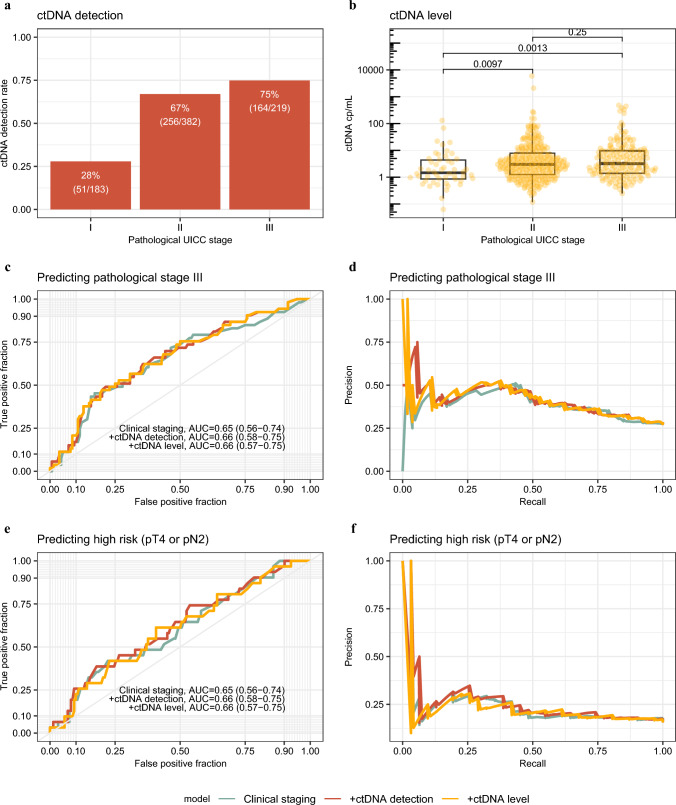
Prediction of pathological stage. **a** Percentage of patients testing pre-operative circulating tumor DNA (ctDNA) positive by pathological Union for International Cancer Control (UICC) stage in the training cohort (*n* = 784). **b** ctDNA level in ctDNA-positive samples by pathological UICC stage (*n *= 471). Levels were compared using a Wilcoxon signed rank test and *P*-values annotated. **c**–**f** receiver operating characteristic (ROC) curve (c+e) and precision-recall curve (d+f) for prediction of pathological UICC stage (c+d) or high-risk disease (e+f) on the validation dataset using clinical factors (green), clinical factors and ctDNA detection (positive vs negative; red), or clinical factors and ctDNA level (undetected vs low level [<3 copies/mL] vs high level [≥3 copies/mL]; yellow). Area under the curve (AUC) and 95% confidence interval annotated for each model

First, we investigated the utility of pre-operative ctDNA in predicting final pathological staging when compared with a clinical prediction model. Here we focused on the ability of ctDNA to identify patients with stage III disease (pT1–4 N1–2), in whom adjuvant chemotherapy would be indicated according to current international guidelines. A significant association between pre-operative ctDNA and pathological stage was identified, where both the proportion of patients with positive ctDNA and the absolute values of ctDNA were significantly higher in patients with stage II and III disease than in those with stage I disease (Fig. 1a, b). However, no significant differences in pre-operative ctDNA were seen between patients with stage II and III disease. A predictive model for pathological stage was developed using clinical characteristics routinely available pre-operatively (see Methods – Prediction models). When compared with this model alone, adding ctDNA did not appear to improve prediction of pathological stage (clinical model alone AUC 0.65 vs clinical model + ctDNA AUC 0.66) (Fig. 1c, d). Current guidelines also define a high-risk subset of patients with stage III disease (pT4 and/or N2), in whom long-course adjuvant chemotherapy should be considered (1). However, even when repeating our analyses to identify this high-risk subgroup, no benefit of adding ctDNA was noted (clinical model alone AUC 0.65 vs clinical model + ctDNA AUC 0.66) (Fig. 1 e, f).

**Fig. 2:**
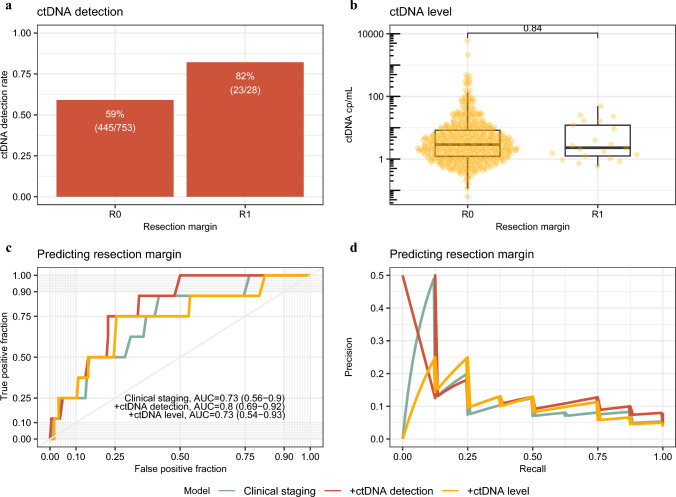
Prediction of resection margin. **a** Percentage of patients testing pre-operative circulating tumor DNA (ctDNA) positive by resection margin in training cohort (*n* = 781). **b** ctDNA level in ctDNA-positive samples by resection margin (*n *= 468). Levels were compared using a Wilcoxon signed rank test and *P*-values annotated. (c+d) Receiver operating characteristics (ROC) curve (**c**) and precision-recall curve (**d**) for prediction of resection margin on validation dataset using clinical factors (green), clinical factors and ctDNA detection (positive vs negative; red), or clinical factors and ctDNA level (undetected vs low level [<3 copies/mL] vs high level [≥3 copies/mL]; yellow). Area under the curve (AUC) and 95% confidence interval annotated for each model

We then investigated the value of ctDNA in predicting resection margin status after surgery. When considering the whole cohort, a greater proportion of patients with incomplete resections had positive pre-operative ctDNA (R0 59% vs R1 82%, *p* = 0.017) (Fig. 2a). However, no statistically significant difference in the absolute values of pre-operative ctDNA was noted between these groups (Fig. 2b). Although the addition of pre-operative ctDNA to a clinical model for prediction of margins status led to a slight improvement, the performance of this model was still only reasonable (clinical model alone AUC 0.73 vs clinical model + ctDNA AUC 0.80) (Fig. 2 c, d).

**Fig. 3:**
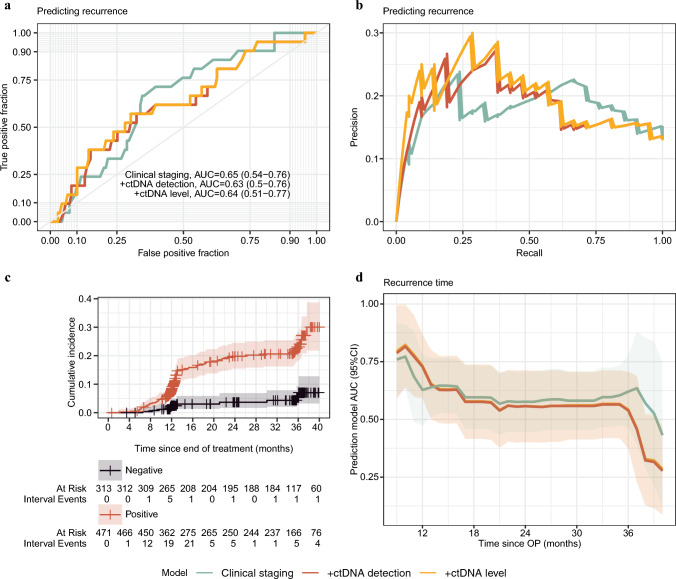
Prediction of recurrence. Receiver operating characteristics (ROC) curve **a** and precision-recall curve **b** for prediction of recurrence on validation dataset using clinical factors (green), clinical factors and circulating tumor DNA (ctDNA) detection (positive vs negative; red), or clinical factors and ctDNA level (undetected vs low level [<3 copies/mL] vs high level [≥3 copies/mL]; yellow). Area under the curve (AUC) and 95% confidence interval annotated for each model. **c** Aalen–Johansen cumulative incidence plot for recurrence stratified for pre-operative ctDNA detection in the training dataset (*n* = 651). **d** AUC for predicting cumulative incidence of recurrence at varying postoperative times (10–40 months) using the same variables as described in **a** and **b**. Shaded area indicates 95% confidence interval

Finally, we investigated whether adding pre-operative ctDNA to a model consisting of routinely used pre-operative clinical variables would improve prediction of post-operative recurrences. Initially a logistic regression model for predicting recurrence status was built using only pre-operative clinical variables (see Methods – Prediction models). Adding pre-operative ctDNA status or ctDNA level did not significantly improve prediction of post-operative recurrence status (clinical model alone AUC 0.65 vs clinical model + ctDNA AUC 0.63) (Fig. 3 a, b). Closer inspection of the resulting odds ratios (ORs) from the multivariable model (Supplementary Figure 1) revealed that, although ctDNA status did not improve the overall prediction of the model, pre-operative ctDNA (OR 3.67 [95% confidence interval (CI) 1.94–7.51], *p* < 0.001) and cN stage (cN1 OR 1.79 [95% CI 1.05–3.07] *p* = 0.033; cN2 OR 2.16 [95% CI 1.03–4.40] *p* = 0.037) were the only variables contributing independently and significantly to prediction of recurrence.

As recurrence is a time-dependent outcome, we also used a used a Fine–Gray competing risk regression model, with death as competing event, to investigate whether the cumulative incidence of recurrence differed for pre-operative ctDNA-negative and ctDNA-positive patients. Patients with positive pre-operative ctDNA had a significantly higher 3-year cumulative incidence of recurrence at 23% (95% CI 18–28) than ctDNA-negative patients (5.3% [95% CI 2.6–9.4]; *p* < 0.001) (Fig. 3c). To explore the predictive strength of pre-operative ctDNA relative to the routinely available preoperative clinical variables, a Fine–Gray competing risk regression model, with death as the competing event, was initially built using pre-operative clinical variables alone and then with the addition of ctDNA status or level. Both the clinical model alone and the two models including ctDNA showed greater power to predict early (<12 months) rather than late recurrences (Fig. 3d). Though not significant, adding ctDNA to the clinical model led to nominally higher AUC values for early recurrence prediction (at 10-months post-operatively, clinical model alone AUC 0.77 vs clinical model + ctDNA AUC 0.82; at 12-months post-operatively, clinical model alone AUC 0.63 vs clinical model + ctDNA AUC 0.73) (Supplementary Figure 2). A forest plot of the subdistribution hazard ratios of the multivariable Fine–Gray model again showed that ctDNA (OR 3.41 [95% CI 1.81–6.43], *p* < 0.001) and cN-category (cN1 OR 1.88 [95% CI 1.13–3.13] *p* = 0.015; cN2 OR 1.73 [95% CI 0.88–3.39] *p* = 0.110) were the only variables significantly contributing to the recurrence prediction (Supplementary Figure 3).

Supplementary analyses were performed to determine whether ctDNA detection rate varied between the three different detection methods used in this study cohort (Supplementary Figure 4). A slightly higher ctDNA detection rate was noted with the use of three colorectal cancer-specific methylation markers. However, a relatively higher proportion of patients analyzed with this technique had larger tumors and more advanced pathological stage, factors associated with higher levels of ctDNA. After adjusting for these factors, no significant association between detection method and detection rate was found.

## Discussion

To the authors’ knowledge, the current study is the largest to date to investigate the potential of pre-operative ctDNA identifying high-risk patients for neoadjuvant therapy in the context of localized and potentially curable pMMR colon cancer. Although pre-operative ctDNA differed significantly between patients with stage I and more advanced disease, no significant differences between those with stage II and III disease was noted. Similarly, although a greater proportion of patients with R1 margins had positive pre-operative ctDNA, only a slight improvement in the prediction of margin status was seen with the addition of ctDNA to other clinical factors. However, although its addition to other clinical factors did not improve overall prediction of recurrence after surgery, pre-operative ctDNA had the strongest association with recurrence of any pre-operative factors, an association that was most marked in patients developing earlier recurrences.

Patient selection remains a major challenge in establishing a role for neoadjuvant chemotherapy in patients with localized pMMR colon cancer. Previous randomized studies have relied on CT-based risk assessment, whereby patients with cT3/4 tumors with or without suspicion of nodal involvement were eligible for inclusion.^7–9^ There are two major limitations with this strategy. First, the accuracy of CT staging is limited and risks overstaging, and thereby overtreating, patients with low-risk cancers.^13^ This was well demonstrated in both the PRODIGE 22 and the FOxTROT trials, where between 25 and 33% of patients in the control arms were found to have pathological stage I or low-risk stage II cancers despite being classified as high risk after clinical staging. Second, an underlying assumption of these stratification methods is that all patients with high-risk stage II and stage III cancers benefit from adjuvant chemotherapy, which would justify the use of neoadjuvant chemotherapy as an alternative. However, this is not the case. The risks of recurrence in patients with stage II or III disease have gradually reduced since the initial randomized trials of adjuvant chemotherapy.^4^ As a consequence, the proposed benefit of adjuvant chemotherapy has come under increased scrutiny, with convincing arguments being made that most of these patients would be cured with modern-day surgery alone.^20–22^ Indeed, one of the most promising clinical applications of ctDNA lies in improving stratification of patients to adjuvant chemotherapy after surgery, allowing these decisions to be made on the basis of the presence of residual disease rather than pathological stage.^14–16^ In this context, post-operative ctDNA has tremendous potential in avoiding overtreatment of patients with both stage II and III disease, who may have already been cured by surgery alone. These developments not only demonstrate the limitations of current clinical and pathological staging in allocating patients to chemotherapy in the adjuvant setting but also highlight the need for better strategies for patient stratification in the neoadjuvant setting.

Focusing on the specific proposed benefits of neoadjuvant therapy may prove to be a more robust stratification strategy. These benefits lie in either reducing the surgical complexity of the planned resection and preventing incomplete (or R1) resections or in reducing the risks of recurrence and cancer-related death.^23^. R1 resections are not infrequent after potentially curative resection for colon cancer and are associated with significantly increased risks of recurrence and death.^24,25^ These increased risks also seem to persist despite long-course adjuvant chemotherapy.^3^ A threatened surgical margin is already one of the established criteria for allocation of patients with rectal cancer to neoadjuvant therapy, where R1 margins are associated with increased risks of both local and distant recurrence.^26,27^ Increased rates of intact resection margins is one of the potential benefits of neoadjuvant chemotherapy in patients with colon cancer, with the FOxTROT trial finding significantly more R0 resections in the interventional arm (94 vs 89%, *p* < 0.001).^9^ As such, several factors provide a rationale for exploring margin-based risk assessment in patients with colon cancer. However, the utility of pre-operative ctDNA in this context appears limited. Although a greater proportion of patients with R1 margins had positive pre-operative ctDNA, the addition of ctDNA to predictive models for margin status only slightly improved performance. Given advances in image quality, the use of CT to predict margin involvement rather than pathological stage is an alternative strategy. Small studies have shown reasonable accuracy in CT-prediction of positive resection margins, where positive CT margins were also independently associated with recurrence-free survival.^28,29^ However, whether the performance of these assessments is reproducible in larger studies remains to be seen.

Several small studies, summarized in a recent systematic review, have previously investigated the association with pre-operative ctDNA and recurrence after surgery in patients with colon cancer.^30^ However, it is difficult to draw any firm conclusions from these studies given the small sample sizes and the lack of adjustments for other known prognostic factors. These limitations are addressed in the current study, which includes a considerable number of patients with relevant follow-up. Although its addition to a model based on routinely available pre-operative clinical factors did not improve performance, several interesting associations between pre-operative ctDNA and recurrence were noted. Not only were patients with positive ctDNA at increased risk of recurrence but also this association appeared strongest in patients who developed early recurrences after surgery. It can be argued that these patients are precisely those who are most likely to benefit from neoadjuvant therapy, allowing micrometastatic disease to be treated as early as possible while avoiding potential delays in adjuvant therapy because of surgical complications. It is also of interest to note that ctDNA status and cN staging were the only pre-operative factors independently associated with an increased risk of recurrence. Clinical T stage has been shown to be associated with recurrence risk and was the main selection criterion used in previous trials of neoadjuvant chemotherapy.^31,32^ It is also recommended as the main method to select patients with pMMR colon cancers for neoadjuvant chemotherapy by current Danish national guidelines. However, with the inclusion of ctDNA in the current study, no significant association with cT stage and the risk of recurrence was noted. This suggests that ctDNA has greater prognostic value for recurrence after surgery than clinical T stage, the main factor that is currently used to allocate patients to neoadjuvant therapy. Future prospective randomized studies investigating whether ctDNA status, alone or in combination with clinical staging, can improve selection of patients to neoadjuvant therapy would be of great interest.

The authors acknowledge the limitations of the current study. Post-hoc analyses were performed on patient samples collected during the IMPROVE study, in which patients underwent standard surgical resection without neoadjuvant therapy. As such, although the current study’s results can assess the association between ctDNA and surrogate outcomes, no comments can be made on the actual benefit from neoadjuvant chemotherapy in these patients. Furthermore, the association between pre-op ctDNA and recurrence may be affected by other treatment-related factors, which cannot be completely accounted for in this study. Both the analyses investigating the association between margin status and recurrence with pre-operative ctDNA are limited by the small number of patients with R1 margins or who developed recurrence after treatment in the current study. As such, the results of the adjusted and subgroup analyses should be interpreted with caution. Finally, several different methods for ctDNA detection were used in patients included in this study, which could raise concerns about heterogeneity within our cohort. However, no significant association between the method of ctDNA detection employed and detection rate was found, in keeping with the findings of previous studies that have demonstrated that the results of these different techniques are comparable.^18,33^

In conclusion, although ctDNA has significant associations with pathological stage, R1 resections and the risk of recurrence after potentially curative surgery, its utility in improving the stratification of patients with localized pMMR colon cancer to neoadjuvant chemotherapy requires further investigation in the context of prospective randomized trials.

## Supplementary Information

Below is the link to the electronic supplementary material.

Supplementary file1 (PDF 1,986 KB)

Supplementary file1 (PDF 485 KB)

Supplementary file1 (PDF 518 KB)

Supplementary file1 (PDF 487 KB)

## Data Availability

In accordance with Danish law, the data on which the findings of this study are based cannot be made available for sharing.
