# Supplementary material for: Potential of Circulating Tumor DNA in Stratifying Patients with Localized pMMR Colon Cancer to Neoadjuvant Therapy
Source: Ann Surg Oncol. 2026 Apr 3;33(7):6530–9. doi: 10.1245/s10434-026-19539-8 (PMC13242440; doi:10.1245/s10434-026-19539-8)
Supplement: Supplementary file 1 — Supplementary file1 (PDF 1,986 KB) [file 10434_2026_19539_MOESM1_ESM.pdf]

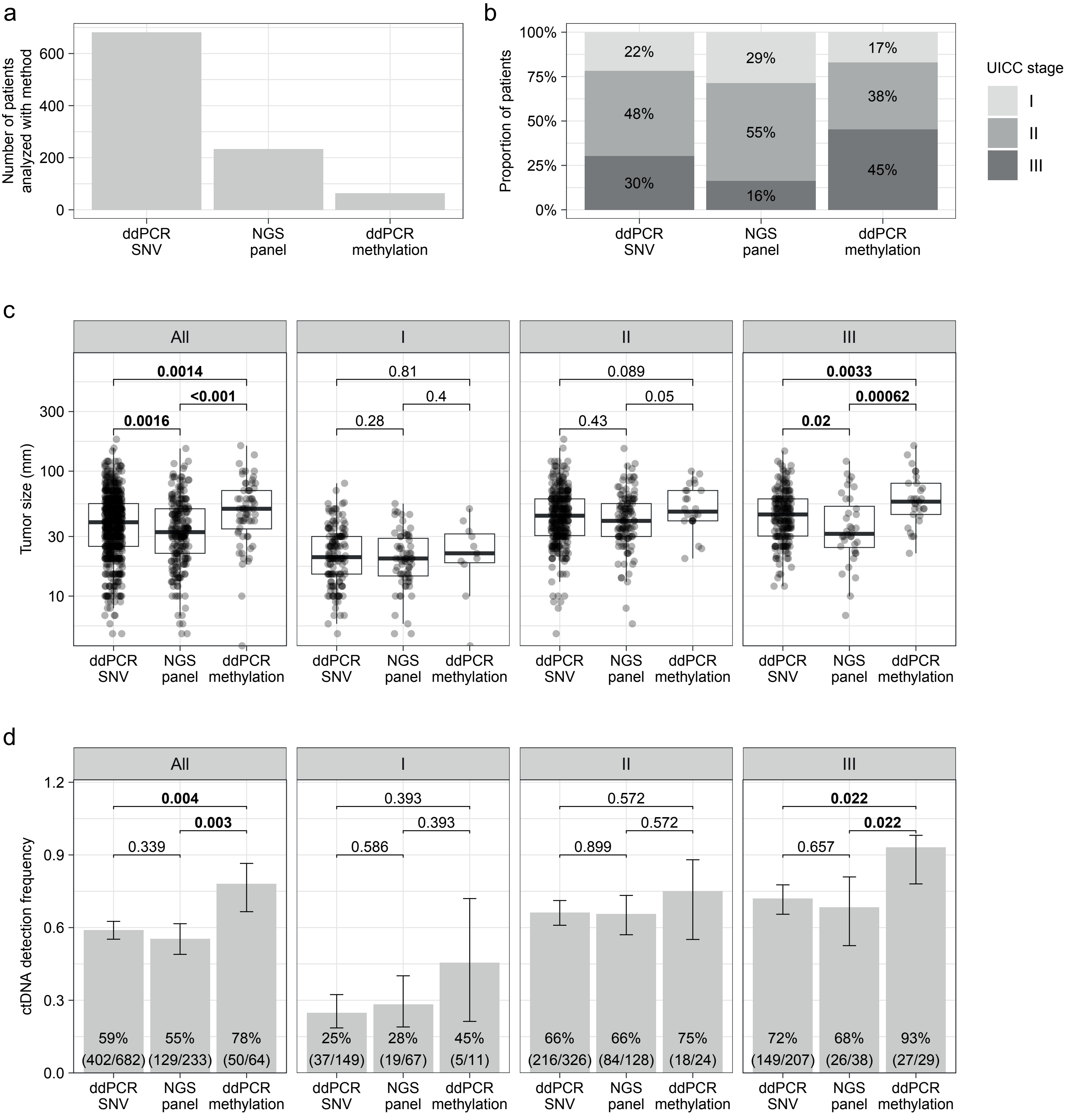

e

Characteristic	OR	95% CI	p−value
Method			
ddPCR SNV	–	–	
NGS panel	1.17	0.83, 1.67	0.4
ddPCR methylation	1.99	1.00, 4.15	0.057
UICC stage			
I	–	–	
II	2.35	1.59, 3.50	**<0.001**
III	3.28	2.11, 5.12	**<0.001**
Tumor size (mm)	1.05	1.04, 1.06	**<0.001**

Supplementary Figure 4 – Comparison of ctDNA detection methods. a) Distribution of methods used across patients. b) Pathological UICC stage distribution among patients analyzed with each method. c) Tumor size (largest diameter in mm) for patients analyzed with each method. d) ctDNA detection frequency by each method for all patients and stage-stratified. Whiskers indicate 95% confidence interval calculated as Wilson's interval. e) Logistic regression analysis for testing ctDNA positive with each different method, when accounting for different stage distribution and tumor size, which are both correlated to ctDNA detection on their own. P-values <0.05 were considered statistically significant and are highlighted in bold.
