# Supplementary material for: Potential of Circulating Tumor DNA in Stratifying Patients with Localized pMMR Colon Cancer to Neoadjuvant Therapy
Source: Ann Surg Oncol. 2026 Apr 3;33(7):6530–9. doi: 10.1245/s10434-026-19539-8 (PMC13242440; doi:10.1245/s10434-026-19539-8)
Supplement: Supplementary file 2 — Supplementary file1 (PDF 485 KB) [file 10434_2026_19539_MOESM2_ESM.pdf]

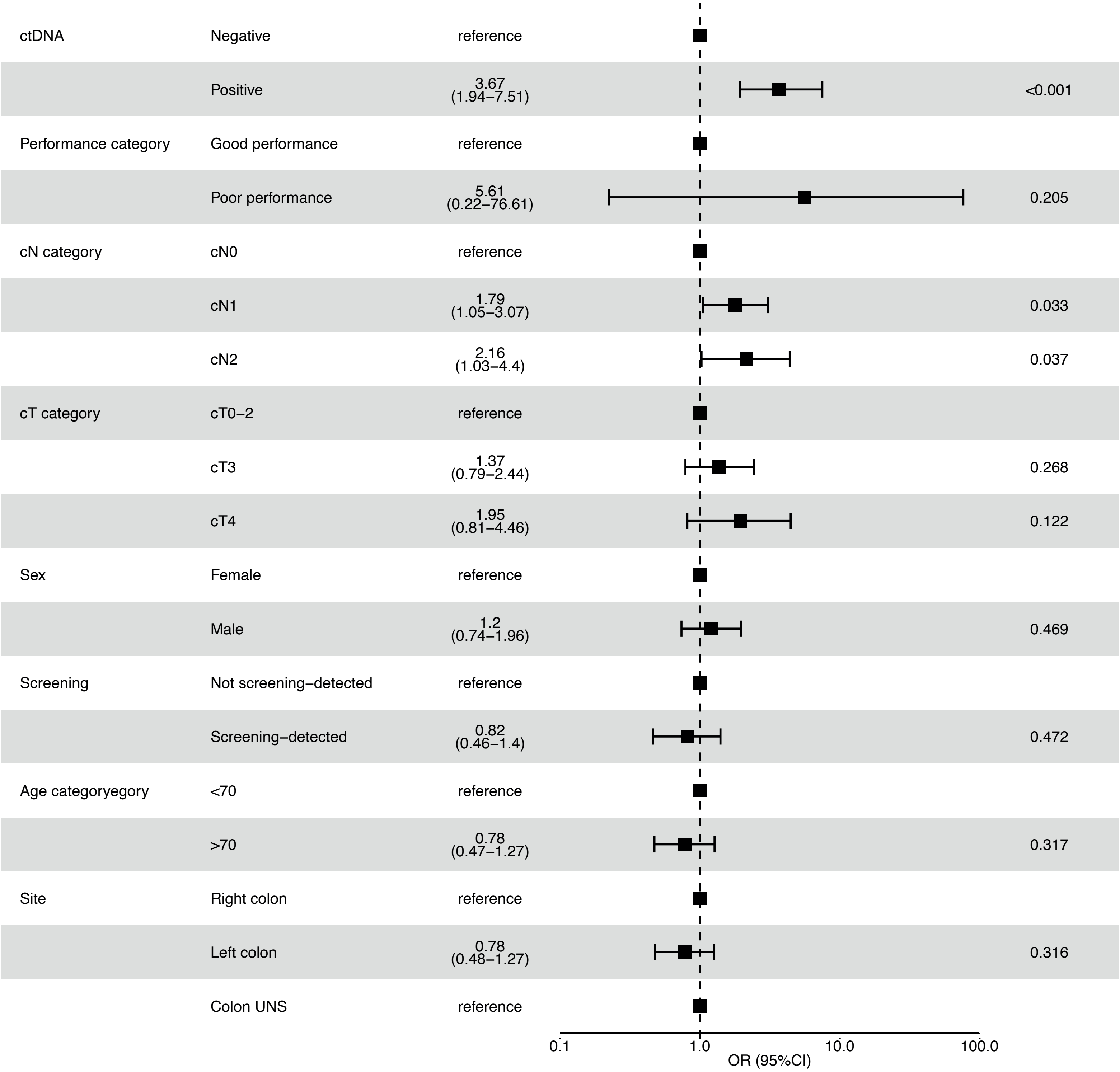

Supplementary Figure 1 – Forest plot depicting multivariable logistic regression model odds ratio (OR) for preoperative ctDNA detection and clinical variables for predicting postoperative recurrence status. OR and 95%CI annotated to the left, p-values annotated to the right.
